# Evaluation of vascular lesions using circulating endothelial cells in renal transplant patients

**DOI:** 10.1111/j.1399-0012.2012.01620.x

**Published:** 2012-04-19

**Authors:** Jiqiu Wen, Jisong Chen, Shu-Ming Ji, Dongrui Cheng, Zhi-hong Liu

**Keywords:** acute rejection, C4d, circulating endothelial cells, kidney transplantation, vascular lesions

## Abstract

Objective: To investigate the correlation between circulating endothelial cells (CECs) and vascular lesions in renal allografts.

Methodology: Sixty-two renal transplant patients were divided into four groups according to biopsy data. CECs were isolated from peripheral blood with anti-CD136-coated immunomagnetic Dynabeads and counted by microscopy during biopsy. CEC numbers were compared in each group, as well as the correlation between CECs and C4d and vascular changes in different groups.

Result: CECs counts were higher in the acute rejection (AR) with endarteritis group than in the normal group (p < 0.01), acute tubular necrosis (ATN) group (p < 0.01) and chronic allograft nephropathy (CAN) group (p < 0.01), there were no difference among ATN, normal and CAN) group (p = 0.587). There was no difference among the normal group without hyaline, normal group with hyaline and CAN with hyaline group. An increasing CECs count was related to C4d-positive AR (p = 0.008; κ score = 0.519) and infiltration of inflammatory cells (p = 0.002, κ score = 0.573) in proximal tubule cells (PTCs). The CECs count decreased after intensive therapy in five patients (p = 0.001).

Conclusion: Elevation of the CEC count in blood was related to endarteritis. Elevation of CEC count was related to C4d deposition and infiltration of inflammatory cells in PTCs.

As surgical techniques and new immunosuppressive agents have developed, the short-term survival of patients who have undergone kidney transplantation has greatly improved. However, refractory acute rejection (AR) after kidney transplantation remains the main cause of short-term graft loss. This has increased the prevalence of chronic allograft nephropathy (CAN), which influences the long-term survival of grafts (1,2).

Several studies have focused on the diagnosis and treatment of AR to find a marker for the prognosis of AR, as well as to find an individual treatment for each patient that can improve survival after AR (3,5). Vascular rejection of renal allografts has been associated with corticosteroid-resistant as well as poor short- and long-term outcome (6,7). Antibody-mediated AR was initially identified by Racusen et al. 1999 and then further described by the same author 2003. Because of the different pathogenesis of each type of AR, they must be distinguished and treated individually 2003.

More than 30 yr ago, Bouvier and colleagues 1970 first reported the presence of non-hematopoietic cells of endothelial origin in the blood of rabbits after endotoxin injection. This was also confirmed by subsequent studies by Hladovec et al. (9,10). Circulating endothelial cells (CECs) have been associated with several pathological conditions that have common vascular injuries (12,13). Also, endothelial cells or endothelial progenitors in the circulation can “home” to sites of ischemia (14,15) as well as play a part in the formation of thrombotic neointima and angiogenesis on vascular prosthetic surfaces in vivo (16,17). Identification of the origins of CECs and blood endothelial outgrowth may facilitate the use of these cells in the clinical diagnosis. Also, measurement of CECs is useful in antineutrophil cytoplasmic antibody (ANCA)-associated small vessel vasculitis 2003. Woywodt et al. reported that CECs are a novel marker of cyclosporine-induced endothelial damage in renal transplant patients 2003. The number of CECs in patients with acute vascular rejection was elevated, and the authors concluded that CEC number was a novel marker of endothelial damage in renal transplantation 2003. There was also report disclosed that an increase in circulating endothelial cells was found to predict the development of cardiovascular and vascular events 2005.

Vascular injury in renal allografts can be assessed by renal allograft biopsy. Intimal arteritis and fibrinoid necrosis are signs of vascular rejection 1999. Inflammatory cells infiltrating into proximal tubule cells (PTCs) have also been associated with antibody-mediated rejection 2003. Intimal thickening in patients with CAN has also been documented 2005. The relationship between CECs and such changes was unclear until now.

The present study was designed to analyze the relationship between CECs and vascular injury in renal allografts and to find a non-invasive marker for vascular injury in renal allografts.

## Materials and methods

### Ethical approval of the study protocol

The study protocol was approved by the Ethical Committee of Jinling Hospital. All patients provided written informed consent to be included in the study.

### Materials

M-450 Dynabeads were purchased from Dynal (Oslo, Norway). Anti-CD 146 antibodies were obtained from Biocytex (Marseille, France). All other reagents were of the highest grade commercially available.

### Patients and control subjects

Sixty-two subjects who had undergone renal transplantation were selected in this study. These patients were hospitalized and underwent renal biopsies at the Renal Transplantation Center of the Research Institute of Nephrology in Jinling Hospital from November 2006 to December 2007. Eighteen healthy volunteers were used so that a normal range of CECs was available. These healthy subjects were selected from the staff of the Research Institute of Nephrology.

All patients were diagnosed according to histological changes in renal allograft biopsies according to the criteria of Banff 07. They were then initially separated into four groups: AR (n = 25); acute tubular necrosis (ATN) (n = 6); normal allograft (n = 18); and CAN (n = 13) 2005. The AR group was then subdivided into the acute antibody-mediated rejection (AAMR) group (n = 13) and T-cell-mediated rejection (TCMR) group (n = 12). The AR group with endarteritis group (n = 12) was specially analyzed in this study. The normal group was defined as patients with a protocol biopsy with normal renal function and normal histological changes. The selected CAN group had the characteristics of intimal thickening accompanied by interstitial fibrosis or tubular atrophy 2005.

### Renal histological examination

Ultrasound guided percutaneous biopsy was performed on each transplant patient. Formalin-fixed tissue was embedded in paraffin using standard procedures. Sections (thickness, 2 μm) were stained with hematoxylin/eosin (H&E), periodic acid-Schiff (PAS), silver methenamine and Masson's trichrome for microscopic pathological diagnoses. For immunofluorescence analyses, renal tissues in optimum cutting temperature (OCT) compound were snap-frozen and maintained in liquid nitrogen. Immunofluorescent staining was carried out on 3-μm cryostat sections using fluorescein isothiocyanate (FITC; Dako, Copenhagen, Denmark)-labeled rabbit anti-human immunoglobulin (Ig) G, IgA, IgM, complement (C)3, C4, and C1q (Dako, Carpinteria, CA, USA). All samples were evaluated by two pathologists who were blinded to the CEC data.

C4d staining was carried out on frozen tissue using an indirect immunofluorescence technique with a primary affinity-purified monoclonal antibody (mouse anti-human; dilution, 1:50; 1.5-h incubation at room temperature; Quidel, San Diego, CA, USA) and a FITC affinity-purified secondary rabbit anti-mouse IgG antibody (1:20; 40-min incubation at room temperature). Positive staining was defined as reported by the Banff 07 2005: C4d0 (0%), C4d1 (1–10%), C4d2 (10–50%) and C4d3 (>50%).

### Isolation and counting of CECs

Isolation of CECs was carried out by immunomagnetic separation after an antibody incubation step according to previously reported and validated methodology 2003. Three milliliters of ethylenediaminetetraacetic acid (EDTA) blood from patients with renal transplantation and from healthy volunteers after obtaining their informed consent was collected for isolation of CECs. Anti-endothelial cell monoclonal antibody (anti-CD146)-coated M-450 Dynabeads were stored at 4°C for a maximum of four wk. Blood from study subjects and healthy controls was obtained by venipuncture. After careful rotation of the tube, 1 ml blood was mixed with 1 ml isolation buffer (phosphate-buffered saline [PBS], 0.1% bovine serum albumin [BSA], 0.1% sodium azide and 0.6% sodium citrate) at 4°C. Samples were mixed in a head-over-head mixer for 30 min at 4°C and separated using a Dynal MPC-1 Magnetic Particle Concentrator (Dynal, Oslo, Norway). The sample was washed with buffer four times inside the magnet at 4°C. Between each washing procedure, the sample was flushed ten times with buffer in a 100-μL pipette. The cell-bead suspension was then dissolved in 200 μL buffer. Cells were counted using a Nageotte chamber. Endothelial cells were larger than other blood cells, had a well-delineated round or oval cell shape, and carried > 5 beads (Fig.). Various concentrations of fresh human endothelial cells from the umbilical vein were diluted in the blood of healthy volunteers to serve as positive controls.

**Figure 1:**
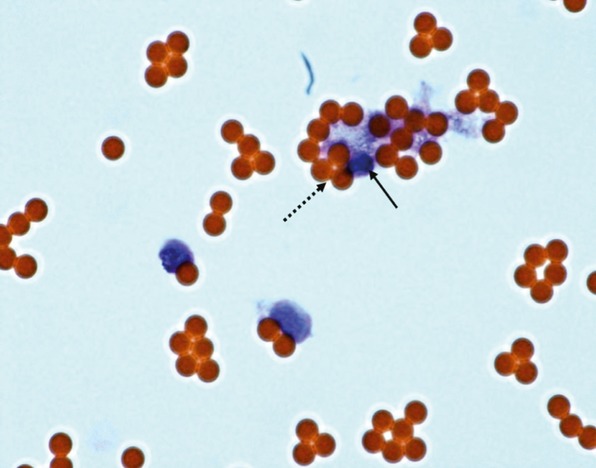
Circulating endothelial cells (CECs) detected by magnetic beads. (HE, ×400, blue arrow: magnetic beads; red arrow: CECs).

### Statistical analyses

Data are mean ± standard deviation. Significant differences between two groups were analyzed using the χ^2^ test. Concordance between two groups was evaluated using Fisher's exact test. All p values were two-sided. p < 0.05 was considered significant. Analyses were carried out using the SPSS version 13.5 statistical package (SPSS, Chicago, IL, USA).

## Results

### Demographic information and clinical characteristics

Sixty-two renal transplant patients were separated into four groups according to the histology of allograft biopsy. Demographic information and clinical characteristics are shown in Table.

**Table 1:** Demographic and clinical characteristics of the four main study groups

	AR (n = 25)	ATN (n = 6)	Normal (n = 18)	CAN (n = 13)
Age (yr)	39.6 ± 10.5	42.8 ± 15.0	39.5 ± 9.0	40.5 ± 12.4
Females/males	15/10	4/2	10/8	3/10
Primary kidney disease				
CGN/other	16/9	4/2	12/6	9/4
Hemodialysis/CAPD	21/4	5/1	16/2	11/2
PRA I (≥10%)	1	0	0	0
PRA II (≥10%)	4	0	0	2
SCr (mg/dL)	2.5 ± 2.2	3.3 ± 2.3	1.0 ± 0.27	2.4 ± 1.0
Oliguria (n)	4	0	0	0
Fever (n)	3/25	0	0	0
Period from surgery (days)	3–368	3–100	14–180	360–860

AR, acute rejection; ATN, acute tubular necrosis; CAN, chronic allograft nephropathy; CGN, chronic glomerular nephritis; CAPD, continuous ambulatory peritoneal dialysis; PRA, plasma rennin activity; SCr, serum creatinine.

### CEC count in different vascular injury groups

The CEC count in different vascular injury groups is listed in Table. Vascular injury included: endarteritis in the AR group; hyaline arteriolar thickening in the normal renal function group; and chronic hyaline arteriolar thickening in the CAN group and ATN group. The CEC count was highest in the endarteritis group. The difference in CEC count between the other groups was not significant (Table; Fig.). We also analyzed the CEC count among three groups (Table).

**Figure 2:**
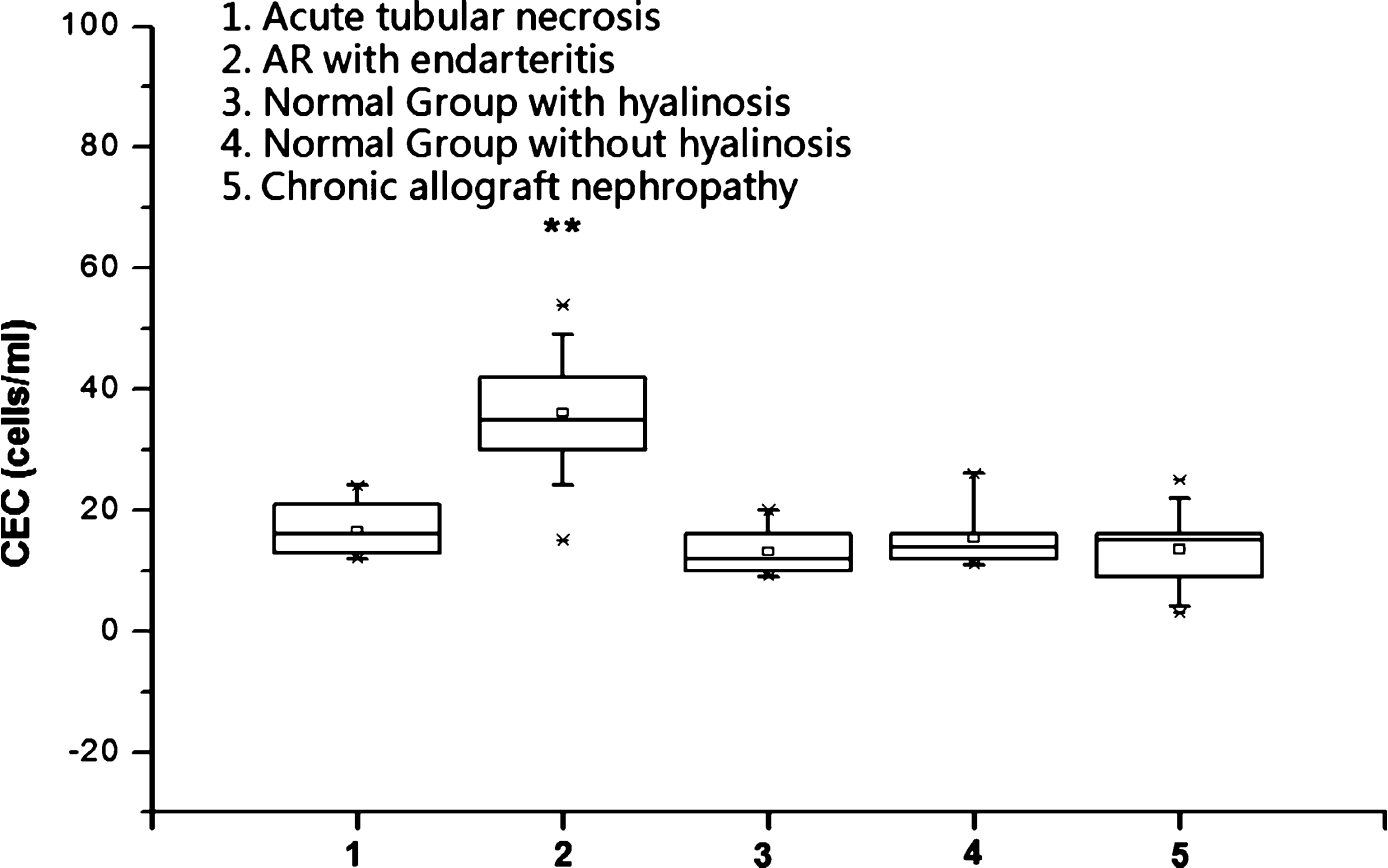
Circulating endothelial cells count in different vascular injury groups. **According to Banff 07. AR: acute rejection; AVR: acute vascular rejection; ATN: acute tubular necrosis; CAN: chronic allograft nephropathy.

**Table 2:** Effect of vascular injury on CEC number

	CEC count (/μL)
AR with endarteritis (n = 12)	36.2 ± 11.1
ATN (n = 6)	16.7 ± 4.8
Normal group without hyaline arteriolar thickening (n = 9)	15.4 ± 4.6
CAN (n = 13)	13.5 ± 6.4
Normal group with hyaline arteriolar thickening (n = 9)	13.2 ± 4.0

AR, acute rejection; ATN, acute tubular necrosis; CAN, chronic allograft nephropathy; CEC, circulating endothelial cells.

### CEC count in different types of AR groups

To identify the relationship between CEC count and AR, we further analyzed the CEC count in different types of AR. C4d deposition in PTCs was considered to be a marker for antibody-mediated rejection. The criteria for C4d deposition was stated in Banff 2007. The C4d-positive group was all C4d3 according to Banff 2007. The CEC count in the AR group was higher than that of the normal group (p < 0.01). The CEC count in the C4d-positive group was higher than that of the C4d-negative group (p < 0.01; Table; Fig.). The CEC count in the C4d-negative group was also higher than that of the normal group (p < 0.01).

**Figure 3:**
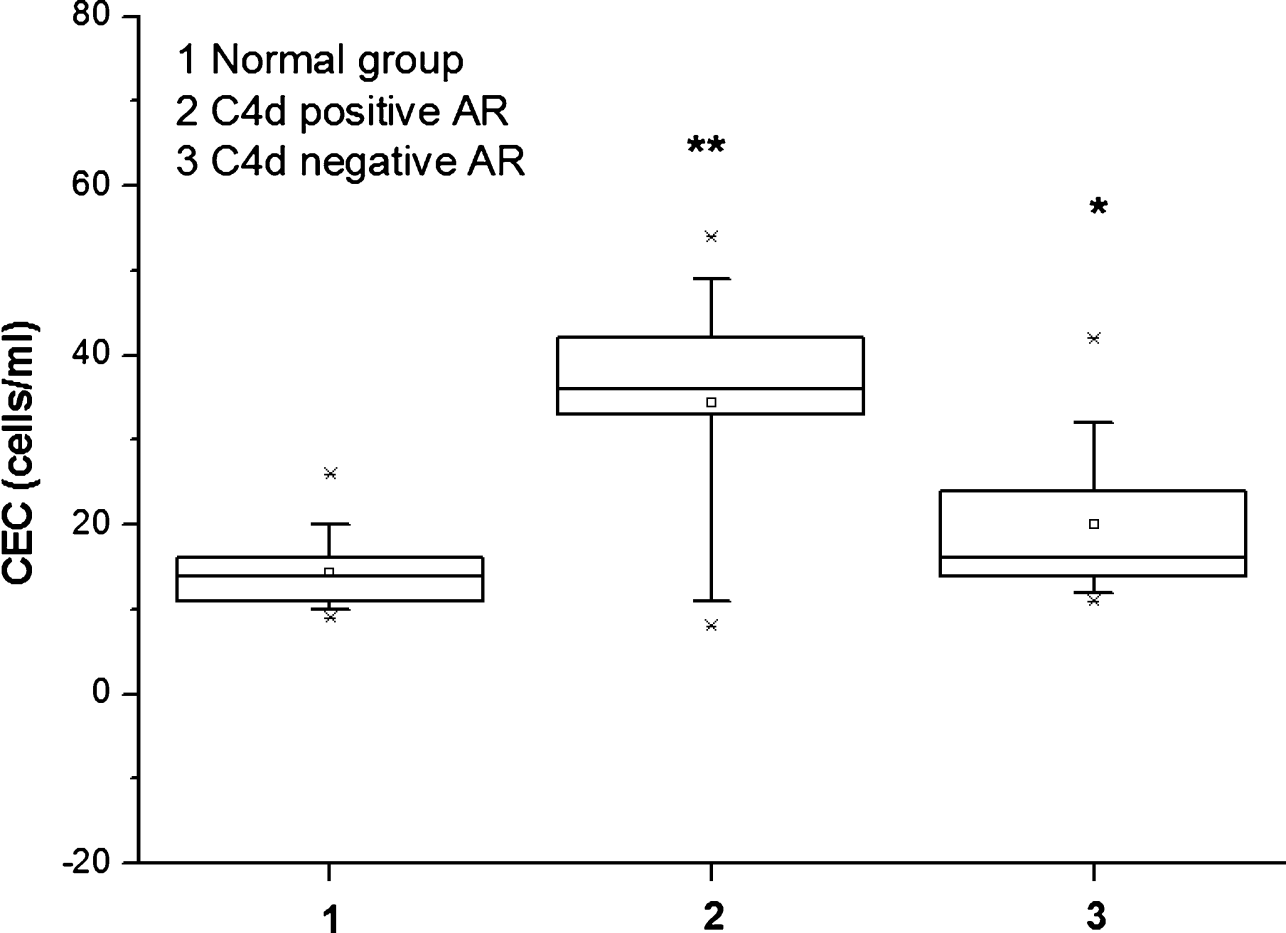
Circulating endothelial cells (CEC) count in different types of acute rejection. * The CEC count in the C4d-positive group was higher than that of the C4d-negative group **According to Banff 07 criteria.

**Table 3:** Effect of acute rejection on circulating endothelial cells (CEC) number

	CEC count (/μL)
C4d-positive AR (n = 13)^a^	34.5 ± 13.7
AR (n = 25)	27.7 ± 13.6
C4d-negative AR (n = 12)^a^	20.0 ± 9.3
Normal group	14.3 ± 4.3

AR, acute rejection.

^*^The CEC count in the C4d-positive group was higher than that of the C4d-negative group According to Banff 07 criteria.

### Relationship between increasing numbers of CECs with C4d-positive cells and inflammatory cells in congested peritubular capillaries

According to the range of CECs in the healthy group and normal group, we considered a CEC count ≥24 CECs/μL as indicating that the number of CECs was increasing. We further analyzed the relationship between increasing numbers of CECs with the number of C4d-positive cells: there was a significant correlation between the two factors (p = 0.028; κ score = 0.437). The presence of inflammatory cells in congested peritubular capillaries was considered to reflect changes in acute humoral rejection. We also analyzed the correlation between the number of inflammatory cells in congested peritubular capillaries and increasing numbers of CECs in the AR group: there was a significant correlation between the two factors (p = 0.002; κ score = 0.573).

### Pathological characteristics and short-term prognosis

To evaluate the relationship between CEC count and pathological characteristics and short-term outcome, we initially divided patients in the AR group into those with a CEC count ≥24/μL and those with a CEC count <24/μL group. We then compared the pathological characteristics and short-term outcome between the two groups. The mean prevalence of glomerulitis, mononuclear cell interstitial inflammation, and tubulitis was compared between the two groups according to Banff 07 criteria. C4d deposition in PTCs, intimal arteritis, and mononuclear cell interstitial inflammation in PTCs were compared as was corticosteroid resistance and graft loss at one yr. Only the prevalence of intimal arteritis was significantly different between the two groups. Corticosteroid resistance and graft loss at one yr was higher in the CEC count ≥24/μL group(Table). The sensitivity and specificity of CEC number > 24 for AR with endarteritis was 83.3% and 69.8%.

**Table 4:** Pathological characteristics and short-term prognosis

	CEC count ≥24/μL group (n = 14)	CEC count <24/μL group (n = 11)	p
Glomerulitis	1.50 ± 0.41	1.60 ± 0.60	NS
Mononuclear cell interstitial inflammation	1.61 ± 0.65	2.25 ± 0.62	NS
Tubulitis	1.48 ± 0.49	1.75 ± 0.75	NS
C4d deposition in PTCs			
Positive/negative	10/4	4/7	NS
Intimal arteritis			
Yes/no	10/4	2/9	<0.05
Mononuclear cell interstitial inflammation in PTCs			
Yes/no	11/3	6/5	NS
Intimal arteritis	5	1	<0.05
Corticosteroid-resistant	8	3	<0.05
Graft loss at one yr	3	1	<0.01

CEC, circulating endothelial cells; PTC, proximal tubule cells.

### Changes in CEC count in subjects with acute vascular rejection before and after effective treatment

Five AR patients suffered endarteritis two wk after transplantation. These AR patients were corticosteroid-resistant and received 3–5 rounds of immunoadsorption. The immunosuppressive protocol was tacrolimus combined with mycophenolate mofetil and prednisone. The renal allograft recovered gradually after intensive immunosuppressive therapy. CEC number also decreased to within the normal range as the function of the renal allograft recovered (Fig.).

**Figure 4:**
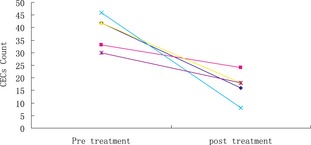
Change in circulating endothelial cells count in five acute rejection patients who received immunoadsorption as well as tacrolimus combined with mycophenolate mofetil and prednisone.

## Discussion

Over the past 30 yr, CEC numbers have been measured in normal individuals and patients with various diseases (22,23). However, these reports are diverse not only because of the different diseases studied, but also because of different methods of isolation and detection (16,24). In 1991, George et al. 1989 unequivocally demonstrated CECs in whole blood using an endothelial cell-specific antibody. Subsequently, several research teams identified CECs in whole blood using endothelial cell-specific monoclonal antibodies.

Damage to endothelial cells is the hallmark of acute vascular rejection, which is an important predictor of graft loss. Nevertheless, endothelial damage and cell death do not necessarily lead to scarring and loss of vascular function. Instead, repopulation of endothelial leaks by recipient stem cells has recently been documented in renal transplant recipients who have previously sustained acute vascular rejection 2003. A continuing interplay between vascular damage and repair has therefore been postulated. This concept mandates that damaged endothelial cells undergo detachment from the basement membrane at some point of the disease process. Putative mechanisms of detachment and factors that protect against it have been reviewed 1991.

An increased number of CECs in acute vascular rejection has been reported 2003. The present study confirmed this finding. The CEC count in patients with AR with endarteritis was highest in the AR group (Fig.). However, the new Banff 07 criteria emphasize that C4d deposited in PTCs is considered to be a marker of antibody-mediated rejection; this type of rejection leads to poor outcome. Therefore, we compared the CEC count between the C4d-positive group and C4d-negative group and further analyzed the correlation between increasing numbers of CECs and C4d deposition in PTCs in the AR group. We found a correlation between these two factors. We also evaluated the relationship between increasing numbers of CECs and monocyte infiltration around PTCs. We found these two factors to be correlated. This finding indicated that injury to the vessel endothelium of the graft probably plays an important part in antibody-mediated rejection. The mechanism of this phenomenon should be investigated further.

Hyalinization of arteries is common in renal allografts, which was related to hypertension and calcinuren inhibitor nephrotoxicity. This change has also been associated with injury to endothelial cells 2001. We evaluated CEC numbers in the group with hyaline arteriolar thickening in the normal group with normal function and compared it with CEC numbers with no hyaline arteriolar thickening the normal group. We also compared CEC numbers with hyaline arteriolar thickening in the CAN group. The results showed no significant difference among the three groups. One study revealed that cyclosporine can increase the number of CECs in transplant patients compared with those in healthy subjects 2003. In the present study, all patients received calcineurin inhibitors, and CEC number was also higher than that in the healthy group (data not shown). This observation could be explained by this effect (19,28). Therefore, hyaline arteriolar thickening does not lead to the increase in the number of CECs. Therefore, in contrast to the increasing number of CECs in the AR group with endarteritis as described before, we concluded that only acute injury to endothelial cells can lead to an increase in the number of CECs in the peripheral circulation. When recovery from such acute injury begins, the CEC number decreases but the vessel does not completely recover (Fig.).

Graft endarteritis was considered to be a characteristic of T-cell-mediated AR according to Banff 97 and Banff 07 criteria. Endarteritis has good correlation with C4d deposition in PTCs (p = 0.003, κ score 0.601). Antibody-mediated rejection also required the activation of T cells. This led to complement activation, which caused allograft injury, but a mixed type of rejection was also noted. Therefore, T-cell-mediated and antibody-mediated rejection cannot be completely separated in practice, and treatment for such patients should be tailored to the individual.

Patients with an increasing number of CECs in the AR group had a high prevalence of corticosteroid resistance and poor short-term outcome. This phenomenon might be explained by an increasing number of CECs being related to endarteritis and C4d deposition in PTCs. Banff 97 guidelines suggest that being C4d-positive can be considered to be a form of corticosteroid-resistant AR, and that the short- and long-term prognosis is poor (21,29-31). Work from our institution confirmed this. Tacrolimus combined with mycophenolate mofetil can effectively treat C4d-positive AR in the short term, but the predictive value of long-term survival should be studied further 1998.

We did not evaluate the origin of CECs. According to the present study and other reports, we hypothesized that the CECs originated from the donor because increasing number of CECs were related to injury of the endomembrane of the vessel of the allograft. This question could be answered by gene sequencing these CECs.

In summary, we revealed that increasing CEC number was related to acute injury to the endomembrane of the renal allograft. The highest CEC count was related to endarteritis and decreased with recovery from the injury caused by endarteritis. CEC number was also related to the C4d-positive AR and the presence of inflammatory cells in congested peritubular capillaries (which also supported the notion that antibody-mediated rejection was related to injury to the endomembrane). CEC number was not related to hyaline arteriolar thickening and chronic vascular injury in renal allografts. An increasing number of CECs can be used as a predictor of poor short-term outcome of AR of renal allografts.
